# Pharmacokinetic Comparison of Ginsenosides between Fermented and Non-Fermented Red Ginseng in Healthy Volunteers

**DOI:** 10.3390/pharmaceutics14122807

**Published:** 2022-12-15

**Authors:** Myeong-Bae Shin, Sung-Ah Kim, Sooyoung Lee, Wang-Seob Shim, Kyung-Tae Lee, Seung-Kwon Lee, Sung-Vin Yim, Bo-Hyung Kim

**Affiliations:** 1 Department of Regulatory Science, Graduate School, Kyung Hee University, Seoul 02447, Republic of Korea; 2 Department of Clinical Pharmacology and Therapeutics, Kyung Hee University Medical Center, Seoul 02447, Republic of Korea; 3 Department of Life and Nanopharmaceutical Sciences, Graduate School, Kyung Hee University, Seoul 02447, Republic of Korea; 4 Kyung Hee Drug Analysis Center, College of Pharmacy, Kyung Hee University, Seoul 02447, Republic of Korea; 5 Department of Biomedical and Pharmaceutical Sciences, Graduate School, Kyung Hee University, Seoul 02447, Republic of Korea; 6 Department of Pharmaceutical Biochemistry, College of Pharmacy, Kyung Hee University, Seoul 02447, Republic of Korea; 7 Ginseng Biotech Research Team, ILHWA Co., Ltd., Guri-si 11933, Republic of Korea; 8 Department of Biomedical Science and Technology, Graduate School, Kyung Hee University, Seoul 02447, Republic of Korea; 9 East-West Medical Research Institute, Kyung Hee University, Seoul 02447, Republic of Korea

**Keywords:** fermented red ginseng, pharmacokinetics, ginsenoside absorption, compound K, compound Y

## Abstract

Fermentation of red ginseng (RG) produces fermented red ginseng (FRG), thereby increasing the relative amount of downstream ginsenosides, including compound Y (CY), F2, Rh2, compound K (CK), compound O, protopanaxadiol (PPD), and protopanaxatriol (PPT). These downstream ginsenosides have beneficial pharmacological effects, and are easily absorbed by the human body. Based on these expectations, a randomized, single-dose, two-period, crossover clinical trial was planned to compare the pharmacokinetic characteristics of seven types (Rb1, CY, F2, CK, Rh2, PPD, and PPT) of ginsenoside components after FRG and RG administration. The safety and tolerability profiles were assessed in this clinical trial. Sixteen healthy Korean male subjects were administered 6 g of FRG or RG. All ginsenosides except Rb1 showed higher systemic exposure after FRG administration than after RG administration, based on comparisons of ginsenoside C_max_ and area under the concentration–time curve (AUC) between FRG and RG. CK, the main ginsenoside component produced during the fermentation process, had 69.23/74.53-fold higher C_max_/AUC_last_ after administration of FRG than RG, and Rh2 had 20.27/18.47-fold higher C_max_/AUC_last_ after administration of FRG than RG. In addition, CY and F2 were detected in FRG; however, all plasma concentrations of CY and F2, except in one subject, were below the lower limit of quantification in RG. There were no clinically significant findings with respect to clinical laboratory tests, blood pressures, or adverse events. Therefore, regular administration of FRG may exert better pharmacological effects than RG.

## 1. Introduction

Ginseng has long been a popular medicinal food in Eastern and Western countries. *Panax ginseng*, also known as Asian or Korean ginseng, is the most widely studied and commonly used ginseng. Ginsenosides, the major active components of *Panax ginseng*, have various beneficial effects, including antioxidant, anti-inflammatory, and anti-apoptotic activities [1,2]. These beneficial effects contribute to the neuroprotective effects on the central nervous system in neurodegenerative diseases [1,3]. Ginsenosides act on lipid homeostasis, positively regulating high-density lipoprotein levels, and negatively regulating low-density lipoprotein and triglyceride levels [4,5]. In addition, a systematic review of in vitro and in vivo studies reported that ginsenosides regulate glycolipid metabolism, and increase leptin sensitivity and insulin resistance [6]. These favorable effects on glycolipids, leptin sensitivity, and insulin resistance suggest an alternative treatment for complex diseases such as metabolic syndrome and cardiovascular disease [7]. Moreover, ginsenosides have anti-cancer effects in vitro and could improve the quality of life or reduce fatigue symptoms in patients with cancer [8]. All these beneficial effects of ginsenosides could prevent the development and progression of various diseases or relieve patients’ symptoms, although some clinical studies have reported negative findings.

**Figure 1:**
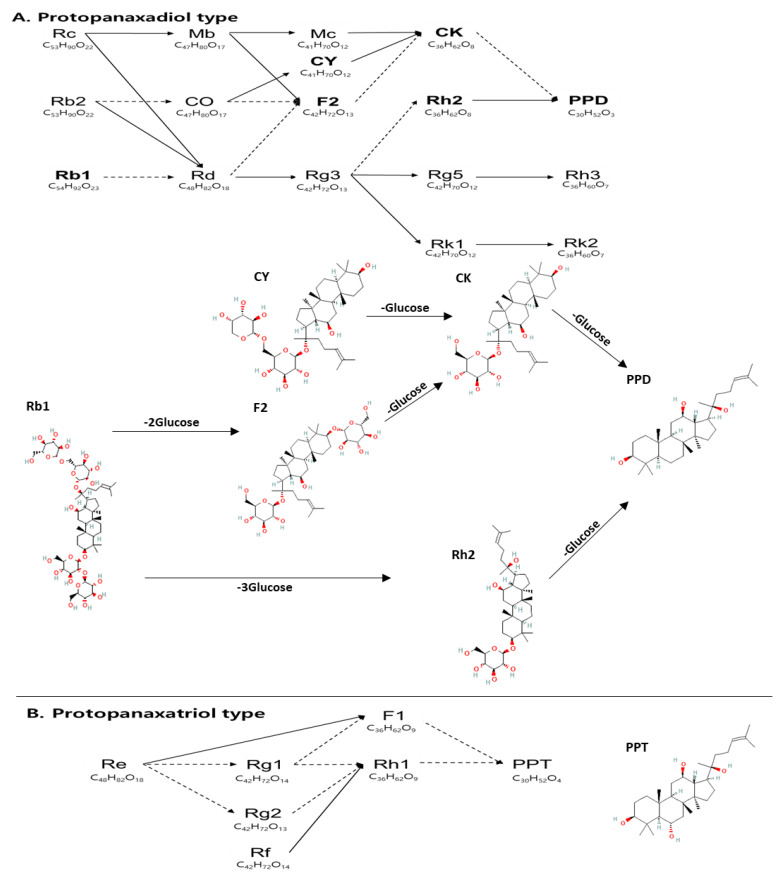
Metabolic pathway of ginsenosides. Ginsenosides are composed of protopanaxadiol (PPD) and protopanaxatriol (PPT) types. Rc, Rb2, Rb1, Mb, CO, Rd, Mc, CY, F2, Rg3, CK, Rh2, Rg5, Rk1, PPD, Rh3, and Rk2 are assigned to the PPD type, and Re, Rg1, Rg2, Rf, F1, Rh1, and PPT are assigned to the PPT type. Dashed lines indicate metabolic conversion by microflora in the human intestinal tract [https://www.koreascience.or.kr/article/JAKO200930858709274.pdf] accessed on 2 September 2022. All structures of analyzed ginsenosides are from PubChem [https://pubchem.ncbi.nlm.nih.gov/] accessed on 19 November 2022. CO: compound O; CY: compound Y; CK: compound K.

Ginsenosides are divided into protopanaxadiol (PPD) and protopanaxatriol (PPT) types according to the hydroxyl group or hydrogen at C_6_ on the structure, corresponding to dammarane-type ginsenosides. Dammarane-type ginsenosides, characteristic components of ginseng, have various pharmacological effects on the central nervous system and cardiovascular system [9]. PPD-type ginsenosides, having a hydrogen at C_6_, consist of Rc, Rb1, Rb2, compound Y (CY), F2, compound K (CK), and PPD, whereas PPT-type ginsenosides, having a hydroxyl group at C_6_, include Re, Rg1, Rf, Rh1, and PPT [Figure 1]. PPD-type ginsenosides have diverse pharmacological effects, such as antioxidant, anti-obesity, and anticancer effects in vitro and in vivo [10,11,12]. PPD-type ginsenosides have several beneficial pharmacological effects such as lipid and antioxidant control [13].

To increase the ginsenoside content of PPD and PPT, fresh ginseng (FG) is converted to red ginseng (RG) by drying and steaming, which decreases the molecular weight and polarity of ginsenosides, especially upstream ginsenosides such as Rb1, Rb2, Rc, and Re, through demalonylation, acetylation and hydrolysis during steaming [14]. Therefore, the relative amount of downstream ginsenosides in RG is higher than that in FG. Meanwhile, the contents of malonyl ginsenosides (Rg1, Rb1, Rb3, Rc, Rd, Rb2) decreased during the steaming process. However, the relative amounts of upstream ginsenosides (Rb1, Rb2, Rc), except Re, are not significantly different between FG and RG [15]. These findings result from the poor absorption of upstream ginsenosides in the intestine; high glycosylation makes the absorption of these ginsenosides difficult [16]. Thus, to improve the absorption of ginsenosides in the human body, a fermentation process was used. This process proceeds via the interaction of ginseng with microorganisms and enzymes. Microorganisms produce various enzymes that can change the structure of ginsenosides by cleaving glucose moieties, and the types of ginsenosides converted are different depending on the microbial strains [17]. Likewise, in the case of enzyme fermentation, the types of enzymes produced differ depending on the type of enzyme present [17]. For example, β-glycosidase is effective in producing CK, and cellulose is used to produce Rh3 [17]. Therefore, deglycosylation of ginsenosides can be actively carried out by fermentation using both intestinal microorganisms, and various enzymes. Accordingly, this fermentation process increases the relative amounts of downstream ginsenosides, that is, CY, F2, CK, and Rh2, which are less polar and have lower molecular weights than upstream ginsenosides.

Considering the fermentation process, the fermentation of red ginseng, that is, fermented RG (FRG), is expected to show the beneficial effects of ginsenosides compared to RG because of the high absorption of these compounds. To test this hypothesis, this study was designed to evaluate and compare the pharmacokinetics of seven ginsenosides (Rb1, CY, Rh2, CK, F2, PPD, and PPT) after oral administration of FRG and RG in healthy volunteers. In addition, tolerability and safety profiles were assessed in a clinical study.

## 2. Materials and Methods

### 2.1. Materials

Both FRG and RG extracts were provided by the Central Research Center of ILHWA Co., Ltd. (Guri-Si, Republic of Korea). Extracts were prepared using the following procedure. Dried RG (1 kg) was extracted with 10 L of ethanol and concentrated using a vacuum concentrator. FRG extract was prepared by fermenting RG with enzymes and *Lactobacillus* spp. This fermentation process converts diol ginsenosides, such as Rb1, Rc, Rb2, and Rd, into smaller ginsenosides, such as CK, CY, compound O, PPD, and PPT, to promote the absorption of ginsenosides in the body.

Ginsenoside content was analyzed using HPLC (Waters Corporation, 34 Maple Street, Milford, MA 01757, USA) equipped with a Waters Atlantis C18 column (4.5 × 250 mm). The mobile phases were water (A) and acetonitrile (B). The injection volume was 10 µL with a gradient as follows: 0–5 min, 20% B; 5–20 min, 23% B; 20–25 min, 30% B; 25–30 min, 40% B; 30–35 min 50% B; 35–60 min, 85% B. The flow rate was 1 mL/min and the column oven temperature was 40 °C. RG extracts contained 1.16 mg/g Rg1, 3.16 mg/g Rb1, 2.66 mg/g Rc, 1.88 mg/g Rb2, 1.57 mg/g Rd, 2.09 mg/g Rg3, 0.21 mg/g Rh2, 0 mg/g CK, 0.14 mg/g CY, 0 mg/g PPD, 0.07 mg/g PPT, and 0 mg/g F2. FRG extracts contained 0 mg/g Rg1, 0 mg/g Rb1, 0 mg/g Rc, 0.13 mg/g Rb2, 0 mg/g Rd, 0 mg/g Rg3, 0 mg/g Rh2, 12.69 mg/g CK, 3.02 mg/g CY, 0 mg/g PPD, 1.15 mg/g PPT, and 2.24 mg/g F2 (Table 1).

### 2.2. Study Design

This study was designed as a randomized, open-label, single-dose, 2-sequence, 2-period crossover study with a washout period of 2 weeks to compare the pharmacokinetic parameters of ginsenosides in RG and FRG. All subjects underwent a screening test for enrollment in the current study, and the enrolled subjects were randomly allocated into one of two sequence groups: sequence 1, and sequence 2. The subjects in sequence group 1 were administered 6 g of FRG extract with 200 mL water in the first period, and 6 g of RG extract with 200 mL water in the second period, after a washout period of 2 weeks from the first administration. Subjects in sequence group 2 were administered 6 g of RG extract with 200 mL water in the first period, and then 6 g of FRG extract with 200 mL water in the second period, after the same washout period. During each period, for groups 1 and 2, subjects were hospitalized at the clinical trial center of Kyung Hee University Hospital at 5 PM the day before FRG or RG administration. They received a standardized dinner and fasted, except for drinking water, until FRG or RG administration. The following day, subjects were administered FRG or RG at 8 AM according to the assigned sequence group, and were not allowed to eat until 1 PM. All participants were provided lunch and dinner by the study staff. They were discharged at 2 PM the day after dosing with FRG or RG, and visited the clinical trial center at 8 AM the day after discharge. To assess pharmacokinetic parameters, blood samples (9 mL) were serially collected into heparinized tubes just before (0 h) the dose of FRG or RG, and after the dose of FRG or RG at the following time points: 0.5, 1, 2, 3, 4, 5, 6, 7, 8, 10, 12, 14, 24, and 30 h during hospitalization, and 48 h at the outpatient visit. Blood samples were placed into heparinized tubes and centrifuged immediately at 3500× *g*, 10 min, and −4 °C. Plasma was stored at −70 °C.

The study protocol was approved by the Institutional Review Board of Kyung Hee University Hospital (IRB number: KHUH 2021-09-067-003, Seoul, Republic of Korea). All procedures were performed in accordance with the principles of the Declaration of Helsinki, and the Korean Good Clinical Practice guidelines.

### 2.3. Study Subjects

Subjects were healthy Korean male volunteers aged 19–45 years with body weight (kg) of 55.0–90.0 and body mass index (BMI, kg/m^2^) of 18.0–27.0. All participants were recruited from the clinical trial center of Kyung Hee University Hospital (Seoul, Republic of Korea), and signed a written informed consent form.

### 2.4. Tolerability and Safety

Throughout this study, safety and tolerability profiles were monitored using the following examinations: adverse events (AEs), blood pressure, body temperature, clinical laboratory tests, and physical examination. The AEs were recorded by spontaneous reports from the subjects and evaluated by the investigator with regard to intensity, duration, and relationship of AEs with administration. Blood pressure and body temperature were measured before administration and at 2, 4, 8, 14, 24, 30, and 48 h after administration of FRG or RG. Clinical laboratory tests and physical examinations were performed before FRG or RG administration and discharge.

### 2.5. Bioanalysis

CY, CK, PPD, PPT, and Rh2 were analyzed as previously described [18]. An Agilent 1200 series (Agilent Technologies, Santa Clara, CA, USA) or Shimadzu Nexera X2 (Shimadzu, Tokyo, Japan), and Applied Biosystems MDS SCIEX API 4000 Triple Quadrupole Mass Spectrometer (SCIEX, Framingham, MA, USA) with an ESI source in the negative ion mode, were used. Data were analyzed and processed using the Analyst 1.6.2 program (SCIEX, Framingham, MA, USA). Rb1 and F2 were analyzed using Elute UHPLC (Bruker, Billerica, MA, USA) and an EVOQ Elite triple quadrupole mass spectrometer (Bruker, Billerica, MA, USA) with an ESI source in positive ion mode. Data were acquired and processed using the Analyst 1.6.2 program (SCIEX, Framingham, MA, USA), and Bruker Daltonic MS Workstation 8.2.1 (Bruker, Billerica, MA, USA). Ginsenosides standards (Rb1, Rh2 and C-Y) were provided by the Ambo Institute (Daejeon, Republic of Korea). Ginsenosides standards (F2, CK, PPD and PPT) were provided by Ace Enzyme (Kyunggi-do, Republic of Korea). Digoxin, diphenhydramine hydrochloride, formic acid, ammonium acetate, and dimethyl sulfoxide were purchased from Sigma Aldrich (Saint Louis, MO, USA). Methanol, acetonitrile, ethyl acetate, and methyl tertiary butyl ether were purchased from J.T. Baker (Philipsburg, NJ, USA) and purified water for HPLC analysis was obtained using AQUA max^®^ by Younglin (Kyunggi-do, Republic of Korea) water purification system. The final concentrations of the calibration standards were 0.5, 1, 2, 5, 10, 25, and 50 ng/mL, and those of the QC samples were 1.5, 7.5, and 40 ng/mL. The final concentrations of the CK calibration standard were 5, 50, 250, 500, 1000, 1500, and 2500 ng/mL, and those of the QC samples were 15, 1200, and 2000 ng/mL. The human plasma samples were taken out of the −70 °C freezer and thawed at room temperature for the bioanalysis. All of the analytical procedures were conducted according to the guidelines of the bioanalytical method, the Korean Ministry of Food and Drug Safety guidelines, which contain validation processes for selectivity, linearity, accuracy, precision, recovery, matrix effect and stability.

### 2.6. Pharmacokinetic and Statistical Analysis

Pharmacokinetic parameters for the FRG and RG groups were calculated using a non-compartmental analysis method using Phoenix WinNonlin 8.3 (Certara USA Inc., Princeton, NJ, USA). The maximum drug concentration (C_max_) and time to achieve C_max_ (T_max_) were obtained directly from individual datasets, including plasma concentration-time profiles. The area under the concentration–time curve (AUC) was calculated by applying the linear-up/log-down trapezoidal rule for non-compartmental analysis. AUC_last_ was calculated from 0 to the actual time of the last measurable concentration above the LLOQ, based on the individual dataset of actual concentrations. The AUC from 0 to 24 h (AUC_24_) was calculated using the individual concentrations, where the values below the LLOQ were replaced by zero.

The demographic characteristics, pharmacokinetic parameters, and safety profiles were explained using descriptive statistics. The logarithmically transformed C_max_, AUC_last_, and AUC_24_ values were compared between the FRG and RG groups using a linear mixed-effect model, which included the fixed effects of sequence, period, and treatment with a random effect of subjects. From these mixed model results, the geometric mean ratios (GMR) with confidence intervals (*p*-values) for C_max_ and AUC of FRG, with respect to those of RG, were obtained by the exponentiation of coefficients for the treatment effect. These GMR values were interpreted as the “fold” of C_max_ and AUC of FRG, compared to those of RG. T_max_ was compared between FRG and RG using the Wilcoxon signed-rank test.

## 3. Results

### 3.1. Subjects

Sixteen healthy Korean male volunteers who met the eligibility criteria participated in the study. The demographic characteristics expressed as mean ± standard deviation of the 16 subjects are as follows: age, 25.69 ± 3.88 years; height, 171.71 ± 3.28 cm; weight, 65.31 ± 5.01 kg; BMI, 22.17 ± 1.85 kg/m^2^. One subject belonging to the sequence 2 group completed this study; however, after period 1, the data were excluded because RG-related foods were taken during the washout period. Finally, the pharmacokinetic results of this study were analyzed in 15 subjects who were administered FRG or RG.

### 3.2. Pharmacokinetic Parameters of Ginsenosides Rb1, F2, Compound Y, Rh2, Compound K, PPD, and PPT

**Figure 2:**
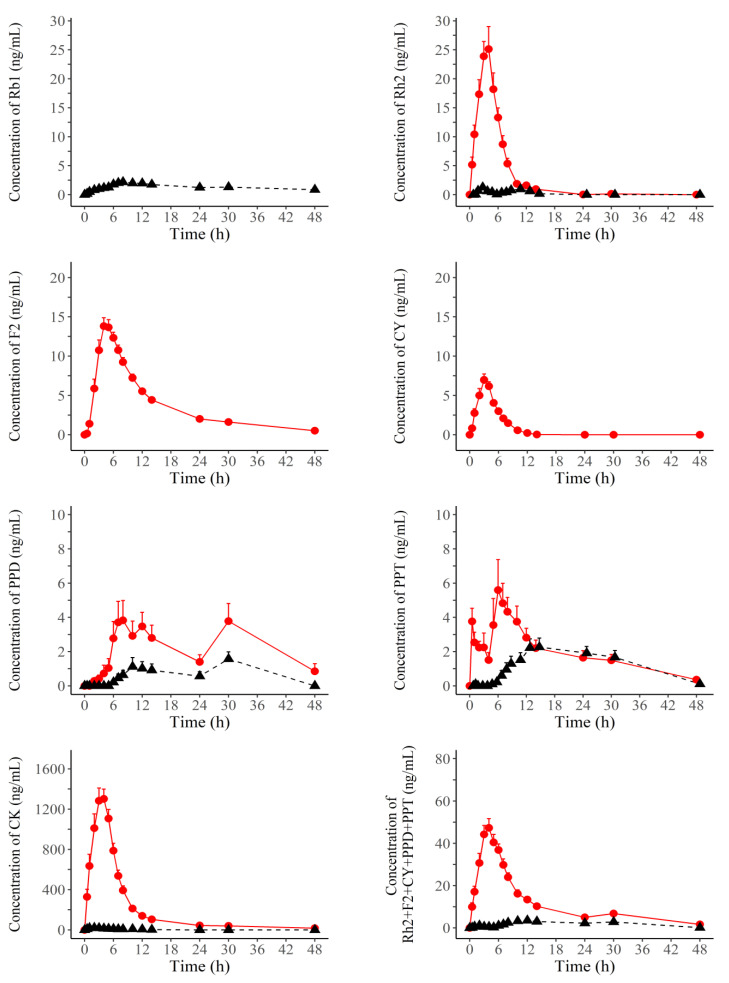
Mean plasma concentration kinetics of ginsenosides after receiving a single dose of 6 g of fermented red ginseng (red filled circles and red solid line) or red ginseng (black filled triangles and black dashed line).

**Figure 3:**
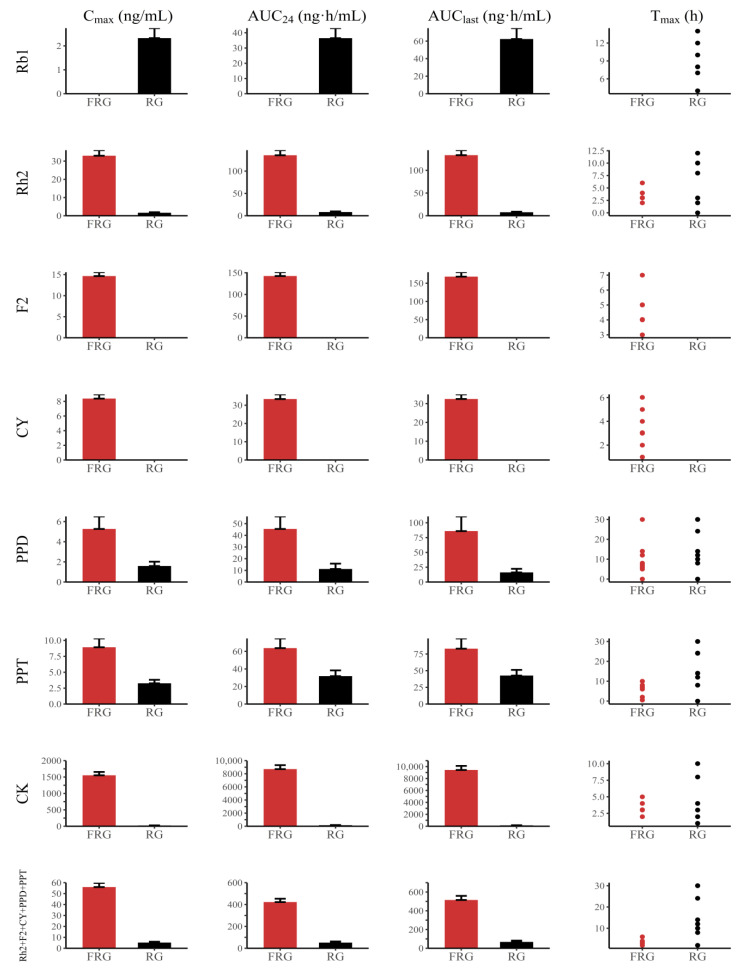
Pharmacokinetic comparisons of C_max_, AUC_24_, AUC_last_, and T_max_ between fermented red ginseng (FRG) (red circles) and red ginseng (RG) (black circles).

The time–mean plasma concentration curves of individual ginsenosides and total ginsenosides in individuals after administration of FRG and RG are shown in Figure 2. The pharmacokinetic parameters of the individual and total ginsenosides after administration of FRG and RG are shown in Table 2 and Figure 3. The C_max_ (ng/mL), AUC_24_ (ng·h/mL), and AUC_last_ (ng·h/mL) of all ginsenosides are shown as mean ± standard deviation and T_max_ (h) as median (min, max). The C_max_, AUC_24_, and AUC_last_ of all analyzed ginsenosides, including total ginsenosides, were significantly different between FRG and RG, except for Rb1, F2 and CY; all comparisons of C_max_, AUC_24_, and AUC_last_ between FRG and RG were statistically significant at *p* < 0.001, except for AUC_24_ and AUC_last_ comparisons of PPD (*p* = 0.033, 0.003) or PPT (*p* = 0.037, 0.029).

Plasma concentrations of Rb1 for FRG, and those of CY and F2 for RG could not be evaluated because these concentrations were below the LLOQ, except for the four consecutive concentrations of F2 for one subject. Therefore, the C_max_, AUC_24_, AUC_last_, and T_max_ of Rb1 were calculated only in RG. Likewise, these pharmacokinetic parameters could be evaluteated only in FRG.

For Rh2, and CK, the C_max_, AUC_24_, and AUC_last_ in FRG were higher than those in RG. The C_max_ of Rh2, and CK was 20.27-, and was 69.23-fold higher in FRG than in RG, respectively. The AUC_24_ values of Rh2, and CK were 18.17-, and 64.86-fold higher in FRG than in RG, respectively. The AUC_last_ values of Rh2, and CK were 18.47-, and 74.53-fold higher in FRG than in RG, respectively. The median T_max_ value of CK in FRG was 1 h higher than that in RG. In Rh2, the median T_max_ values were similar between FRG and RG.

Regarding PPD, PPT, and total ginsenosides (Rh2 + F2 + CY + PPD + PPT), C_max_, AUC_24_, and AUC_last_ were higher in FRG than in RG. The C_max_ of PPD, PPT, and total ginsenosides was 2.89-, 2.56-, and 12.41-fold higher in FRG than in RG, respectively. The AUC_24_ values of PPD, PPT, and total ginsenosides in FRG were 2.95-, 1.87-, and 10.8-fold higher, respectively, than those in RG. The AUC_last_ values of PPD, PPT, and total ginsenosides were 4.97-, 1.76-, and 10.17-fold higher, respectively, in FRG than in RG. The median T_max_ values of PPD, PPT, and total ginsenosides in FRG were lower than those in RG.

### 3.3. Tolerability and Safety

In this study, eight cases of AEs were spontaneously reported in 5 of the 16 subjects. There were three cases of diarrhea and one case of abdominal discomfort in three subjects, which were probably related to FRG administration. Additionally, one subject had diarrhea after both FRG and RG administration. The patient who was excluded from the pharmacokinetic assessment had nausea and nasopharyngitis; however, neither AE was related to the FRG or RG extracts. No serious AEs occurred during the clinical study. There were no clinically remarkable findings in the clinical laboratory tests, blood pressure, body temperature, or physical examination.

## 4. Discussion

Ginsenosides enter the systemic circulation through absorption and metabolism in the gastrointestinal tract and liver to exhibit pharmacological effects, such as anti-cancer and anti-oxidant effects. To improve the absorption of ginsenosides into the systemic circulation, the relative amount of downstream compounds among all types of ginsenosides should be large in ginseng extracts. Because the fermentation process of RG, that is, FRG, increases the relative amount of downstream ginsenosides, intake of FRG is expected to increase the absorption of these compounds. This expectation was confirmed in the current study, in which the absorption or exposure to ginsenosides was much higher for FRG than for RG. The difference in systemic exposure was assessed by comparing the C_max_ and AUC values of ginsenosides after FRG and RG administration. In particular, CY, F2, CK, Rh2, PPD, and PPT showed higher systemic exposure to FRG than RG. However, systemic exposure of Rb1 was only observed in RG. This is because Rb1 is converted into downstream ginsenosides during fermentation. Most ginsenosides in FRG were absorbed faster than those in RG in the assessment of median T_max_ values. These findings can be interpreted as taking more time for the transformation of downstream ginsenosides in RG, which has higher contents of upstream ginsenosides than FRG. However, the T_max_ of Rh2 was similar between FRG and RG, and the T_max_ of CK for RG was 1 h lower than that of FRG. Individual comparisons of T_max_ for CK showed that the inter-individual variability of T_max_ for FRG was smaller than that for RG.

Rb1, the most upstream product of the PPD-type ginsenosides, has a high molecular weight (MW) and polarity because of glucose at C20 of the structure, which disturbs the absorption of Rb1 into the body [16]. Rb1 is metabolized to downstream ginsenosides with low MW and low polarity, that is, CY, F2, and Rh2, by deglycosylation of glucose by enzymes during the fermentation process [17]. The effect of fermentation was confirmed by comparing the individual amounts of ginsenosides in RG and FRG. Rb1 was contained in only RG extract, while it was absent in FRG extract. Accordingly, the C_max_ and AUC of Rb1 could be confirmed only after administration of RG extract. These results also suggest that Rb1 is metabolized to downstream ginsenosides during the fermentation process of RG extract.

F2, CY, and Rh2 are produced by the deglycosylation of precursor ginsenosides, such as Rb1 and Rb2. The content of F2, a metabolite of Rb1 and Rb2, was higher in FRG than in RG, and C_max_ and AUC were also considerably higher in FRG than in RG. Considering that the plasma concentration number above LLOQ in RG was four for only one subject, it is speculated that Rb1 in RG would have been metabolized into F2 or Rg3 by the gastrointestinal microbiome or the liver after absorption, and that the metabolic conversion into F2 could be small, or F2 would be rapidly converted to the downstream ginsenosides such as CK and PPD. Similarly, FRG contained higher amounts of CY, a metabolite of Rb2, than RG, and systemic exposure to CY was higher in FRG than in RG. All the plasma concentrations of CY were below the LLOQ after receiving RG. Considering that the content of CY was 0.14 mg/g in RG, most of the CY in RG would have been converted into downstream ginsenosides, such as CK, when it was absorbed into the body.

The contents of CY and F2 in FRG were 3.02 mg/g and 2.24 mg/g, respectively. However, the systemic exposure of CY was relatively small compared to that of F2 in FRG administration; the C_max_ and AUC_last_ of CY were 8.38 ng/mL and 32.47 ng·h/mL, respectively, and the C_max_ and AUC_last_ of F2 were 14.65 ng/mL and 168.07 ng·h/mL, respectively. These findings suggest that Rb2 is more metabolized into F2 than into CY, and that CY is rapidly metabolized into CK during absorption of CY, compared to F2. Meanwhile, the pharmacokinetic findings of Rh2 between FRG and RG were similar to those of F2; the C_max_ and AUC were much higher with FRG administration than with RG administration. These pharmacokinetic differences were the opposite of the difference in the content between FRG and RG; the Rh2 content was higher in RG than in FRG. These results might lead to interpretation that upstream ginsenosides such as F2, within FRG, was mainly transformed into Rh2 by intestinal microflora or microorganisms from fermented food products which were in the provided diet during the study. These findings should be clarified through further studies.Meanwhile, F2 has a protective effect against skin inflammation by inhibiting interleukin (IL)-17A, a major cytokine in skin inflammation, and ROS production in neutrophils [2]. In addition, F2 reduces hair loss by disrupting the expression of TGF-β2-related factors involved in hair loss, and the SCAP-related apoptosis pathway in vitro and in vivo [19]. CY increases the synthesis of procollagen, a protective factor against skin aging in vitro [20]. Rh2 suppresses adipocyte differentiation by inhibiting PPAR-γ, the major transcription factor for adipocyte differentiation in vitro [11], and reduces ischemic brain injury by inhibiting the synthesis of PGE2 in LPS-stimulated RAW264.7 cells [21]. Therefore, FRG administration is expected to show the aforementioned beneficial effects for ginsenosides F2, CY, and Rh2, compared to RG administration.

Among the PPD-type ginsenosides, CK has less polarity and lower MW compared with upstream ginsenosides, leading to easy absorption into the human body. CK is mainly produced from F2 and CY through the fermentation process, which is shown by the difference in CK levels between FRG (12.69 mg/g) and RG (0 mg/g). Additionally, the C_max_ and AUC of CK were much higher after FRG administration than after RG administration. In the case of RG, although the content of CK was not present in RG, it is considered that the CK concentration was measured through the metabolic pathway from Rc, Rb1 and Rb2 to CK. In addition, C_max_ (/g) and AUC (/g), normalized by the amount of FRG in the current study, were compared with the pharmacokinetic results of previous studies, normalized by the administered amount of FRG; the normalized C_max_ and AUC of CK in the current study were significantly higher than those in the previous study of Japanese volunteers. Normalized C_max_ (/g) and AUC_24_ (/g) for the current study were 2039.81 ng/mL and 11,436.7 ng·h/mL, respectively, and normalized C_max_ (/g) and AUC_24_ (/g) of the previous study were 922.22 ng/mL and 5288.89 ng·h/mL, respectively [22]. In particular, unlike previous FRG studies that focused only on the pharmacokinetics of CK, the current study showed that CK was better absorbed than the other ginsenosides (Rh2, F2, CY, PPD, and PPT), comparing the mean AUC value of CK with that of Rh2 + F2 + CY + PPD + PPT [23]. Furthermore, unlike the previous study in which blood samples were collected up to 24 h after administration of FRG and RG, a larger number of blood samples were collected up to 48 h after administration of FRG and RG in the current study [23]. Numerous studies have reported the pharmacological effects of CK. For example, CK promotes the survival of neurons by inducing brain-derived neurotropic factor (BDNF) and neuronal growth factor (NGF), which have neuroprotective activity in mice and rats [16] and have a favorable effect on streptozotocin-induced diabetic rats by activating the PI3K/Akt signaling pathway-related insulin sensitivity [23]. CK increases heme oxygenase-1 (HO-1) with anti-inflammatory activity in mice [24], and exerts anti-cancer effects by promoting caspase-3, which induces apoptosis in vitro [10] and suppresses cell DNA synthesis, inducing cell cycle arrest at the G1 phase [25]. Therefore, regular administration of FRG used in this study is expected to maximize the pharmacological effects of CK.

PPD and PPT are the end products of PPD- and PPT-type ginsenosides, respectively. In addition, both C_max_ and AUC of PPD and PPT were higher in the FRG group than in the RG group. The median T_max_ values of PPD and PPT were were smaller during FRG administration than during RG administration. In particular, the C_max_, AUC_24_, and AUC_last_ of PPD were approximately 3-, 3-, and 5-fold higher, respectively, with FRG administration than with RG administration. These findings resulted from PPT content being higher in FRG than in RG, and more upstream ginsenosides after administration of FRG were being metabolized to PPD, compared to RG; although, PPD content was absent in both FRG and RG. As there are no studies with the same design, it is difficult to directly compare the pharmacokinetics of PPD and PPT between RG and FRG. However, a recent study comparing the pharmacokinetics of RG and black ginseng (BG) showed that the systemic exposure to PPD and PPT was larger with BG administration, than with RG administration, although the systemic exposure between them was not significantly different [26]. This previous study also showed that other intermediates of ginsenosides were easily absorbed into the human body when receiving BG. The findings of previous and current studies suggest that many intermediate metabolites of PPD-type ginsenosides are easily absorbed and rapidly converted to PPD, when receiving FRG. With regard to the total ginsenoside content of Rh2 + F2 + CY + PPD + PPT, FRG contained a higher content of these ginsenosides than RG. In addition, systemic exposure to these ginsenosides was much higher with FRG administration than with RG administration. These findings suggest that large amounts of ginsenosides can be absorbed more easily by FRG administration than by RG administration.

In the current study, AEs were reported in five subjects. Among them, three subjects reported diarrhea after FRG administration, and one subject reported diarrhea after both FRG and RG administration. Additionally, one subject showed abdominal discomfort after FRG administration. In previous studies, five cases of diarrhea were reported after the administration of 3 g of FRG for 24 subjects [27], and two cases of diarrhea after the administration of 3 g of FRG or RG for 24 subjects [28]. In addition, there was one case of diarrhea after the administration of 9 g of RG to 18 subjects [26]. All AEs in the previous and current studies were evaluated as being of mild intensity, and all administrations in the current study were resolved without serious AEs. Therefore, it is expected that the frequency of diarrhea will decrease by consuming FRG at less than 6 g at a time, and repeatedly consuming FRG as a functional food.

## 5. Conclusions

We compared the pharmacokinetics of PPD- and PPT-type ginsenosides between FRG and RG administration in humans. All analyzed ginsenosides, except for Rb1, showed much higher systemic exposure with FRG administration than with RG administration. Furthermore, the systemic exposure of CK in the current study was much higher with FRG administration than with RG administration, based on the finding that the C_max_, AUC_24_, and AUC_last_ of CK were higher in FRG than in RG. These results suggest that regular administration of FRG can exert the pharmacological effects of ginsenosides more effectively than RG. In addition, there are few reports on the pharmacokinetics of CY and F2 in human clinical studies. Thus, the current study is valuable because it provides a pharmacokinetic basis for the investigation of various ginsenosides, including CY and F2.

## Figures and Tables

**Table 1:** The contents of ginsenoside between fermented red ginseng (FRG) or red ginseng (RG).

Ginsenoside	**PPD-Type**										**PPT-Type**	
	Rc	**Rb1**	Rb2	Rd	**Rh2**	**F2**	**CY**	Rg3	**CK**	**PPD**	Rg1	**PPT**
FRG (mg/g)	0	0	0.13	0	0	2.24	3.02	0	12.69	0	0	1.15
RG (mg/g)	2.66	3.16	1.88	1.57	0.21	0	0.14	2.09	0	0	1.16	0.07

Plasma concentrations were evaluated for 7 ginsenosides of Rb1, Rh2, F2, CY, CK, PPD, PPT (bold).

**Table 2:** Pharmacokinetic parameters of ginsenosides after receiving 6 g of fermented red ginseng (FRG) or red ginseng (RG) in healthy volunteers.

Ginsenoside	Parameter	FRG (*n* = 15)	RG (*n* = 15)	GMR (90% CI)
Rb1	C_max_	- ^2^	2.33 ± 1.61	-
	AUC_24_	- ^2^	36.46 ± 25.57	-
	AUC_last_	- ^2^	62.42 ± 48.06	-
	T_max_	- ^2^	8.00 (4.00, 14.00)	-
Rh2	C_max_	32.95 ± 11.88	1.77 ± 1.17 ^4^	20.27 (15.13, 27.17) *
	AUC_24_	135.40 ± 44.43	8.97 ± 5.57 ^4^	18.17 (12.4, 26.62) *
	AUC_last_	133.43 ± 42.65	8.35 ± 4.31 ^4^	18.47 (12.74, 26.78) *
	T_max_	3.02 (2.00, 6.02)	3.00 (2.00, 12.02) ^4^	0.318 ^1^
F2	C_max_	14.65 ± 3.58	- ^3^	-
	AUC_24_	142.45 ± 34.27	- ^3^	-
	AUC_last_	168.07 ± 47.39	- ^3^	-
	T_max_	4.03 (3.00, 7.00)	- ^3^	-
CY	C_max_	8.38 ± 2.22	- ^3^	-
	AUC_24_	33.41 ± 9.82	- ^3^	-
	AUC_last_	32.47 ± 9.44	- ^3^	-
	T_max_	3.02 (1.00, 6.02)	- ^3^	-
PPD ^5^	C_max_	5.65 ± 4.78	1.99 ± 1.62	2.89 (2.04, 4.09) *
	AUC_24_	48.71 ± 41.15	13.94 ± 19.08	2.95 (1.37, 6.33) ^†^
	AUC_last_	92.16 ± 96.45	20.32 ± 25.11	4.97 (2.38, 10.34) ^‡^
	T_max_	12.01 (5.00, 30.00)	27.05 (8.00, 30.03)	0.203 ^1^
PPT	C_max_	8.92 ± 5.38	3.50 ± 2.00 ^4^	2.56 (1.83, 3.57) *
	AUC_24_	63.72 ± 42.77	34.11 ± 24.68 ^4^	1.87 (1.16, 3.01) ^†^
	AUC_last_	83.03 ± 59.44	45.74 ± 32.10 ^4^	1.76 (1.17, 2.65) ^†^
	T_max_	6.00 (0.50, 10.00)	19.04 (8.00, 30.03) ^4^	<0.001 ^1^
CK	C_max_	1553.11 ± 394.16	23.81 ± 12.16	69.23 (55.93, 85.69) *
	AUC_24_	8707.88 ± 2360.72	158.28 ± 125.29	64.86 (51.12, 82.29) *
	AUC_last_	9438.74 ± 2618.68	145.43 ± 106.45	74.53 (59.32, 93.64) *
	T_max_	3.02 (2.00, 5.00)	2.00 (1.00, 10.02)	0.281 ^1^
Rh2 + F2 + CY + PPD + PPT	C_max_	55.98 ± 14.61	5.24 ± 3.09	12.41 (8.83, 17.45) *
	AUC_24_	422.94 ± 118.64	51.74 ± 41.06	10.8 (7.34, 15.89) *
	AUC_last_	515.26 ± 170.16	68.18 ± 53.92	10.17 (6.92, 14.96) *
	T_max_	3.02 (2.00, 6.02)	12.02 (2.00, 30.03)	<0.001 ^1^

Values are expressed as the mean ± standard deviation, except for T_max_, which is expressed as the median (min, max). GMR, geometric mean ratio; C_max_, maximum drug concentration; T_max_, time to achieve C_max_; AUC_24_, area under the concentration–time curve from 0 h to 24 h; AUC_last_, AUC from 0 h to the actual time point with the last concentration > lower limit of quantification (LLOQ). The ^1^ T_max_ values of FRG and RG were compared using the Wilcoxon signed-rank test. Descriptive statistics could not be calculated, because ^2^ all concentrations of Rb1 for FRG, and thoses of ^3^ CY and ^3^ F2 (except of 1 subject) for RG were below the LLOQ. ^4^ The number of pharmacokinetic parameters was 14 for Rh2 and PPT, respectively. ^5^ The number of pharmacokinetic parameters was 14 for FRG, and 12 for RG. All comparisons of C_max_, AUC_24_, and AUC_last_ between FRG and RG were statistically significant at * *p* < 0.001, except for AUC comparisons of PPD or PPT (^†^ *p* < 0.05, ^‡^ *p* = 0.003).

## Data Availability

Owing to ethical and privacy concerns, individual data are shared only on reasonable request.
